# Assessment of the respiratory function of farmers exposed to pesticides in the municipality of Quilombo (state of Santa Catarina, Brazil): relationship between health and occupational protection

**DOI:** 10.47626/1679-4435-2021-551

**Published:** 2021-04-30

**Authors:** Helber Luiz Bombardelli, Mariana Rossetto, Indiamara de Oliveira Flores Dal Magro Silvani, Vinícius José de Oliveira, Cléber Luis Bombardelli, César Augusto França-Abrahão

**Affiliations:** 1 Curso de Fisioterapia, Universidade Comunitária da Região de Chapecó, Chapecó, SC, Brazil; 2 Departamento de Saúde Coletiva, Faculdade de Medicina, Universidade Federal de Uberlândia, Uberlândia, MG, Brazil; 3 Curso de Fisioterapia, Centro Universitário Avantis, Balneário Camboriú, SC, Brazil

**Keywords:** agriculture, occupational diseases, lung volume measures, respiratory system

## Abstract

**Introduction:**

Rural workers are exposed to various occupational risks, especially considering the exposure to pesticides. This exposure can lead to respiratory intoxications being the most frequent complaint by these professionals, which could be associated to the lack or improper use of personal protective equipment.

**Objectives:**

To assess the respiratory function of rural workers exposed to pesticides in the municipality of Quilombo, state of Santa Catarina, Brazil.

**Methods:**

This study was quantitative, observational, and descriptive. Our sample consisted of 31 rural workers aged between 25 and 45 years and divided into 2 age groups; we aimed to assess the effect of the period of exposure to pesticides. The participants answered a questionnaire, followed by a respiratory function assessment including measurements of inspiratory and expiratory muscle strength using a manovacuometer, of peak expiratory flow with a peak flow meter, and of tidal volume with a ventilometer.

**Results:**

The groups presented decreases in respiratory muscle strength, peak expiratory flow, and tidal volume.

**Conclusions:**

The damage to expiratory function observed in the evaluated sample presents, as a main factor, the lack of personal protective equipment use; therefore, education and health strategies are needed to instrumentalize these workers and reduce the development of occupational risks.

## INTRODUCTION

In Brazil, land management dates from the country’s discovery, in 1500, when Brazilian agriculture was determined by colonizers who brought along animal and plant species.^1^ Together with the productive effort and local knowledge of the land by indigenous peoples, an extremely rich agricultural production was developed in this tropical region. In the 1970s, this activity went through a modernization process where primary agriculture was replaced by more modern techniques and practices with the aim of increasing productivity and, by consequence, profitability.^1^

During this transformation, strategies such as the production of chemical agents that accelerate seed germination, plant growth, and protect seeds and/or plants against harmful agents had to be developed. At this moment, pesticides emerged in Brazil as the scientific solution for controlling pests that hit crops and herds, providing the farmer with higher probabilities of a good crop.^2,3^ It is important to note that the use of pesticides has increased in a gradual and uncontrolled manner in Brazil since 2008, when the country became the world’s biggest pesticide consumer even if not the main global agricultural producer.^4^

The excessive use of agricultural pesticides leads to various problems ranging from the environment to the workers’ health, leading to physiological alterations in their respiratory, circulatory, and/or reproductive systems and adding a burden to the Unified Health System (SUS) through hospital admittances and medication treatments that significantly reduce the working lifespan of rural workers.^5^ Moreover, the need for specialized assistance to the rural worker class should be noted, considering a special attention by health care professionals and government agencies through qualitative and quantitative public policies that monitor the work environment. These strategies should be aimed at controlling the duration of the exposure to pesticides, as well as providing instructions for the adequate handling of personal protective equipment (PPE).^4^ Health care professionals must realize that work is an important determinant of health,^6^ since the knowledge of health problems affecting rural workers contributes with the planning of strategies for dealing with specific occupational problems of this population in all levels of care.^6,7^

Among states in the South region of Brazil, Santa Catarina has shown an increase in the number of reported intoxications due to pesticides in recent years. In 2007, 224 (4.5%) intoxications by pesticides were reported; in 2010, 438 (5.6%) cases were reported, and in 2015, 695 (5.89%) cases were reported, according to the Department of Environmental Health and Worker Health Surveillance.^8^ According to the Santa Catarina Toxicological Assistance and Information Center (Centro de Informação e Assistência Toxicológica de Santa Catarina, CIATox/SC), 196,118 human intoxications were diagnosed between 1984 and 2017, of which 13,414 (6.84%) were triggered by pesticide exposure.^9^ In addition, 88% of the cases progressed to death in 2017, as reported by the university hospital of Universidade Federal de Santa Catarina.^10^

In view of this situation and understanding the occupational risks faced daily by rural workers in direct contact with pesticides (mainly through inhalation), we proposed the present study for assessing the respiratory function of rural workers living in the countryside of the municipality of Quilombo, state of Santa Catarina, who are exposed to pesticides during their work activities.

## METHODS

We initially performed informal visitations to rural properties in the municipality of Quilombo in order to bond with the workers, describe our research, invite them to participate in the study, and present the free and informed consent form (FICF), guaranteeing the privacy of their data and their use for exclusively scientific purposes. We thus obtained the approval of the research project by the Research Ethics Committee of Universidade Comunitária Regional de Chapecó, under *ad referendum* No. 136/CEP/2012, in agreement with Resolution No. 196/96/CNS and its complementary resolutions by the National Health Council.

### STUDY DESIGN

This is a quantitative, observational, and descriptive study, comprising a sample of 31 male rural workers aged between 25 and 45 years; this convenience sample was selected based on their professional exposure or contact with pesticides during a period of 5 years or more. Workers were separated into 2 groups: 1) rural workers aged between 25 and 34 years; 2) rural workers aged between 35 and 45 years. The selected exclusion criteria were as follows: workers who were absent from their residences after 2 consecutive visits and those with previous medical diagnosis of respiratory diseases or other disease that could alter our criteria for respiratory function assessment.

### DATA COLLECTION AND ANALYSIS

#### Questionnaire

We constructed a questionnaire for obtaining data regarding life, work, and health conditions of the selected participants. The following information was collected: age, gender, address, most frequently used pesticides, signs of immediate intoxication, PPE use, and ways of spraying (machinery or manual) the agrochemicals. We also verified the existence of pain and discomfort complaints that were unrelated to work and to previously reported diseases. After applying the questionnaire, we collected vital signs and anthropometric measurements via a physical evaluation.

#### Peak expiratory flow (PEF)

Measurement of PEF used a peak flow meter (ASSESS^®^ Meter, Respironics, New Jersey, USA). For performing this test, the worker had to stand up and perform a maximal inspiration. Then, the participant should place the device between the lips, sealing around the mouthpiece to prevent the air from escaping, and completely exhale fast and intensely through the mouth. The test was performed 3 times and the highest result measured by the device was recorded. The reference value for men was obtained through the following equation:

$$\mathrm{PEF}\left(L/\min\right)=295.79\times \mathrm{height}+\left(24.96\times \mathrm{age}\right)-478.24,$$

according to previously published literature.^11,12^

#### Analysis of inspiratory and expiratory muscle strength

Inspiratory and expiratory muscle strength was measured through manovacuometry, which is a simple, fast, and non-invasive test where maximal inspiratory pressure (MIP) and maximal expiratory pressure (MEP) are obtained. For the test, we used a previously calibrated manovacuometer (Comercial Médica^®^). The participant remained seated, with his body forming 90º angles at the hips and knees. He then was instructed to hold the device tightly between his lips to prevent the air from escaping. Then, the participant’s nostrils were closed with a nose clip and the device’s orifice was occluded to prevent the air from escaping.

For measuring the strength of expiratory muscles, participants were required to perform a maximal inspiration, forcing the expiration through the mouthpiece; for verifying the strength of inspiratory muscles, workers were required to perform a maximal expiration, place the device between the lips, and force the inspiration through the mouthpiece. The test was performed 3 times, and all results were recorded. The calculation used for predicting normal values for men was performed through the following equations:

$$\mathrm{MIP}=155.3-0.80\times \mathrm{age};\;\mathrm{MEP}=165.4-0.81\times {\mathrm{age}}^{12}$$

#### Tidal volume analysis

To measure the workers’ tidal volume, we used ventilometry; this technique is responsible for assessing lung mechanics in health care settings. We used a ventilometer (DHD Healthcare^®^) for measuring volumes and pulmonary ventilation, thus determining tidal volume and minute volume of the individuals in each respiratory cycle.

For the ventilometry test, participants were instructed to remain seated, with the body forming 90º angles at the hips and knees. Then, workers should place the mouthpiece between their lips, sealing so as no air could escape, and perform one deep inspiration, expiring through the device. The test was performed only once, and the result was recorded. The calculation used for predicting normality for men was performed according to the following equation:

deadspace×kg×3,

where the physiological dead space is 150 mL.^12^

#### Statistical analysis

Statistical procedures were performed using Microsoft Excel, version 2007, for data entry. Then, SPSS was used for comparing results using a Student’s *t*-test. Categorical variables were expressed as absolute and relative frequencies, while numerical variables were reported as means ± standard deviations. We considered p-values < 0.05 as statistically significant.

## RESULTS

### PHYSICAL AND OCCUPATIONAL CHARACTERISTICS

All rural workers assessed in this study were male. Their age ranged according to the group to which they were allocated: Group 1 comprised 14 individuals with a mean age of 30.5 ± 2.66 years, while Group 2 consisted of 17 workers whose mean age was 42.35 ± 2.05 years.

As seen in Table 1, the fraction of workers exposed to pesticides varied between groups. In both groups, 100% of the workers were exposed to glyphosate (a green label pesticide) and 45% were exposed to Priori Xtra (yellow label); meanwhile, 64% of the individuals in Group 1 were exposed to Cruiser (blue label) and 29% of those in Group 2 were exposed to Decis (blue label). The vital signs (systolic arterial pressure, diastolic arterial pressure, respiratory rate, cardiac rate) and anthropometric data (height and weight) were similar between groups, as shown in Table 1.

**Table 1:** Physical and occupational characteristics of the participants

Age groups	Pesticides	Exposed individuals - n (%)	Vital signs and anthropometric data	Mean ± SD
Group 1 (n = 14); 25-35 years	Glyphosate (green label)	14 (100)	SAP/DAP (mmHg)	124/76 ± 7/6
	Cruiser (blue label)	9 (64)	RR (breaths per minute)	22.7 ± 3.3
	Priori Xtra (yellow label)	10 (72)	CR (beats per minute)	80.8 ± 18.5
			Height (m)	1.78 ± 0.06
			Weight (kg)	82.0 ± 11.6
Group 2 (n = 17); 36-45 years	Glyphosate (green label)	17 (100)	SAP/DAP (mmHg)	123/74 ± 14/12
	Cruiser (blue label)	5 (29)	RR (breaths per minute)	22.0 ± 3.4
	Priori Xtra (yellow label)	4 (24)	CR (beats per minute)	96.2 ± 30.5
			Height (m)	1.74 ± 0.06
			Weight (kg)	78.1 ± 12.1

CR = cardiac rate; DAP = diastolic arterial pressure; n = number of workers; RR = respiratory rate; SAP = systolic arterial pressure; SD = standard deviation.

### PPE USED BY THE WORKERS AND PHYSICAL HEALTH COMPLAINTS RELATED TO WORK

The PPE used by workers included aprons (10%), boots (94%), gloves (35%), overalls (45%), masks (84%), head covers (42%), and face shields (13%); boots and masks were the most frequently used pieces of PPE. Moreover, workers reported physical health complaints related to their occupation, such as headaches (29%), hypoesthesia of the lips (6%), dizziness (6%), and cough (6%), in addition to eye irritation (3%); these results are shown in Table 2.

**Table 2:** Personal protective equipment (PPE) used by workers and physical health complaints related to work

Available PPE	Individuals who used PPE (%)	Physical complaints by workers	Number of complaints (%)
Apron	3 (10)	Headache	9 (29)
Boots	29 (94)	Hypoesthesia of the lips	2 (6)
Gloves	11 (35)	Eye irritation	1(3)
Overalls	14 (45)	Dizziness	2 (6)
Mask	26 (84)	Cough	2 (6)
Head cover	13 (42)		
Face shield	4 (13)		

### RESPIRATORY VARIABLES ASSESSED IN GROUP 1

The results obtained after the analyses of the respiratory variables MIP, MEP, PEF, and tidal volume, compared to the predicted values for gender and age in this population, as well as the statistical analyses employed, are described/represented in table 3.

**Table 3:** Predicted and measured values of respiratory variables considering workers in Group 1 (25-34 years, n = 14)

Variables	Predicted value mean ± SD	Measured value mean ± SD	p-value
MIP (mmH2O)	131.4 ± 2.4	98.6 ± 22.7	< 0.0001
MEP (mmH2O)	141.2 ± 2.4	108.3 ± 14.2	< 0.0001
PEF (L/min)	618.5 ± 23.3	577.8 ± 85.6	< 0.0001
Tidal volume (mL/min)	517.2 ± 123.1	319.2 ± 129.3	< 0.0001

MEP = maximal expiratory pressure; MIP = maximal inspiratory pressure; n = number of workers; PEF = peak expiratory flow; SD = standard deviation. p < 0.05 was considered significant by a Student's t-test.

### RESPIRATORY VARIABLES ASSESSED IN GROUP 2

As proceeded in group 1, the variables were measured, and compared with the predicted values for worker’s gender and age, and the results are represented in table 4, with their statistical analysis.

**Table 4:** Predicted and measured values of respiratory variables considering workers in Group 2 (35-45 years, n = 17)

Variables	Predicted value mean ± SD	Measured value mean ± SD	p-value
MIP (mmH2O)	121.6 ± 2.1	92.9 ± 21.2	< 0.0001
MEP (mmH2O)	131.2 ± 2.1	107.6 ± 5.9	< 0.0001
PEF (L/min)	569.7 ± 20.4	545.3 ± 88.9	< 0.1441
Tidal volume (mL/min)	519.9 ± 83.8	308.1 ± 79.5	< 0.0001

MEP = maximal expiratory pressure; MIP = maximal inspiratory pressure; n = number of workers; PEF = peak expiratory flow; SD = standard deviation. p < 0.05 was considered significant by a Student's t-test.

## DISCUSSION

During the 1960s and 1970s, the rural environment was greatly influenced by modernization, resulting in the development of the so-called productive agriculture and the emergence of “rural businesspeople.”^1^ This modernization led to the beginning of the social organization of rural workers as a class, influencing the first attempts at rural women’s movements for participation in previously male-dominated environments.^1^ Nevertheless, the sample studied here consisted exclusively of men (100%), which corroborates other studies that indicate that at least 75% of rural workers who use some type of chemical compound (such as pesticides) are men.^13,14^

In our sample group, direct exposure to various kinds of pesticides with different toxicity classifications during work activities is a constant reality. Although glyphosate is classified at the lowest toxicity level,^15^ it is noteworthy that 100% of the workers informed having daily contact with this pesticide. Despite this massive exposure to glyphosate, a significant fraction of the workers reported concomitant exposure to other pesticides such as Cruiser and Priori Xtra, which are classified as of average and high toxicity, respectively.^15^ These aspects highlight the need for PPE when handling these chemical agents with the aim of preventing health problems.

In the studied population, PPEs were seldom used, except for boots (94%) and masks (84%); this can be related to an inefficient adjustment of PPE in Brazil, whose tempered and humid climate increases these workers’ negligence regarding PPE use.^7,16^ Moreover, in order to avoid thermal, visual, and respiratory discomfort, workers prefer not to use PPE and expose themselves to occupational hazards, which has also been reported by other researchers.^7,16^

These factors expose workers to high risks of acute intoxications by pesticides. Soares et al.^17^ reported that wearing a mask during this work activity decreases chances of intoxication in 83% and that the chances of intoxication for those who disregard PPE use due to heat are 535% higher than for those who do not use this equipment for other reasons.

Even though it is a green-label pesticide, glyphosate has raised interest among researchers regarding the effects of the exposure to this component due to its widespread global use. In 2011, a literature review by Salazar-López and Madrid^18^ aimed to elucidate the effects of this pesticide in human and animal health, as well as in the soil and aquatic ecosystems.

Regarding human health, studies have shown that glyphosate presents toxicity to placental cells, acts as an endocrine disruptor of aromatase activity, may alter the structure of the cells’ DNA, produce red blood cell lysis, and cause respiratory, gastrointestinal, allergic, dermatological, neurological, and psychological problems even when used within the allowed conditions.^18^

In face of these aspects, the association between the inadequate use of these substances, the toxicity of certain products, lack of PPE use, and the precarity of surveillance mechanisms^19^ demonstrates the vulnerability of rural workers in work/health relationships, as described by Gregolis et al.^20^ This leads to alterations in the homeostasis of various physiological symptoms, especially considering acute intoxications of the respiratory and gastrointestinal tracts, as reported by some authors.^4,7,10^

In this study, during interviews, workers reported physical health complaints of occupational nature, of which headaches were the most common. These complaints are in agreement with previous studies that verified the presence of strong headaches as a recurring symptom in 69%^21^ of the rural workers who were analyzed and in 71% of the studied sample.^22^ On the other hand, rural workers’ vital signs did not present noticeable differences when compared to the normal values, predicted according to current literature.^23^

Data obtained through manovacuometry in the studied groups presented lower values when compared to the literature^12,24^; this was expected since Simões et al.^25^ noted that a physiological loss of strength by respiratory muscles occurs with age, being more evidenced from the fourth decade of life. The measured values in the studied population were thus expected to be altered: their decrease was enhanced due to pesticide exposure.

Senhorinho et al.^26^ studied the prevalence of ventilatory disorders in rural workers exposed to pesticides in the northern region of the state of Paraná, correlating the observed disorders with a predisposition to respiratory intoxication by rural workers. The authors state that a significant decrease (when compared to predicted values) in parameters such as PEF and respiratory volumes is in agreement with obstructive respiratory disorder (ORD) presentations, demonstrating that the exposure to pesticides in this population has led to the development of ORDs, independently of its duration.

The significant decrease in respiratory variables studied in Group 2, except for PEF, corresponds to other studies observed in the literature. Oliveira^27^ demonstrated that pesticide exposure led to a decrease in MIP and MEP; França et al.^28^ proved that 100% of rural workers assessed in their study showed PEF and tidal volume values that were lower than the predicted ones. Buralli^29^ verified, in his sample, a decrease in the respiratory condition of a rural population exposed to pesticides in the municipality of São José de Ubá, state of Rio de Janeiro, while Senhorinho et al.^26^ verified this effect in a population living in northern Paraná.

The fact that no significant difference was observed in the PEF of individuals in Group 2 may be related to the issue that older and more experienced workers are the main PPE users, since according to data published by Senhorinho et al.,^26^ they tend to protect themselves more than younger workers. Moreover, variations in the PEF variable are risk factors for the development of occupational asthma,^30^ which makes Group 2 less vulnerable to developing this disease than Group 1.

Finally, we were able to identify a resistance to the use of PPE by rural workers for various reasons, such as the lack of training for appropriate use and social and environmental conditions. Therefore, the clarification of the risk conditions to which rural workers are exposed, decrease in the direct exposure to pesticides (without PPE), and improvements in work conditions consist in actions that are capable to guarantee worker protection and reduce impacts to their health.

We conclude that it is possible that alterations in respiratory variables found in the studied population are due to the inadequate use of PPE, although most rural workers are aware of their importance; this has been proven to directly impact the workers’ pulmonary health. In addition, more studies are required for developing a way of measuring the respiratory function of rural workers exposed to pesticides, as well as for investigating possible health-promoting actions for this population by interprofessional teams.
